# Comparison of lung microbiota between antineutrophil cytoplasmic antibody-associated vasculitis and sarcoidosis

**DOI:** 10.1038/s41598-020-66178-4

**Published:** 2020-06-11

**Authors:** Shoichi Fukui, Shimpei Morimoto, Kunihiro Ichinose, Shota Nakashima, Hiroshi Ishimoto, Atsuko Hara, Tomoyuki Kakugawa, Noriho Sakamoto, Yoshika Tsuji, Toshiyuki Aramaki, Tomohiro Koga, Shin-ya Kawashiri, Naoki Iwamoto, Mami Tamai, Hideki Nakamura, Tomoki Origuchi, Yukitaka Ueki, Shino Suzuki, Hiroshi Mukae, Atsushi Kawakami

**Affiliations:** 1 Department of Immunology and Rheumatology, Nagasaki University Graduate School of Biomedical Sciences, Nagasaki, Japan; 2 Department of Community Medicine, Nagasaki University Graduate School of Biomedical Sciences, Nagasaki, Japan; 3 Innovation Platform & Office for Precision Medicine, Nagasaki University Graduate School of Biomedical Sciences, Nagasaki, Japan; 4 Department of Respiratory Medicine, Nagasaki University Graduate School of Biomedical Sciences, Nagasaki, Japan; 5 Rheumatic and Collagen Disease Center, Sasebo Chuo Hospital, Sasebo, Japan; 6 Center for Bioinformatics and Molecular Medicine, Nagasaki University Graduate School of Biomedical Sciences, Nagasaki, Japan; 7 Department of Rehabilitation Sciences, Nagasaki University Graduate School of Biomedical Sciences, Nagasaki, Japan; 8 Kochi Institute for Core Sample Research, X-star, Japan Agency for Marine-Earth Science and Technology (JAMSTEC), Nankoku, Japan

**Keywords:** Vasculitis syndromes, Vasculitis syndromes

## Abstract

Microbial involvement in the pathogenesis have been suggested in both antineutrophil cytoplasmic antibody-associated vasculitis (AAV) and sarcoidosis, both of which have lung involvement. However, exhaustive research to assess the bacteria in the lung in AAV and in sarcoidosis have not been performed. We sought to elucidate the distinct dysbiotic lung microbiota between AAV and sarcoidosis. We used 16S rRNA gene high-throughput sequencing to obtain the bacterial community composition of bronchoalveolar lavage fluid (BALF) in patients with AAV (n = 16) compared to patients with sarcoidosis (n = 21). The patients had not undergone therapy with immunosuppressive medication when their BALF was acquired. No difference was observed in α-diversity between patients with AAV and patients with sarcoidosis when using all the detected taxa. We defined the taxa of the oral cavity by using the data of oral microbiota of healthy individuals from the Human Microbiome Project (HMP). The analysis using only oral taxa made the difference in α-diversity between AAV and sarcoidosis clearer compared with those using all the detected taxa. Besides, the analysis using detected taxa except for oral taxa also made the difference in α-diversity between AAV and sarcoidosis clearer compared with those using all the detected taxa. A linear negative relationship between the α-diversity and Birmingham vasculitis activity score (BVAS) was detected in the AAV group. The observed p-value for the effect of the disease groups on the ß-diversity was small while the effect of other factors including sex and smoking status did not have small p-values. By excluding oral taxa from all the detected taxa, we found a cluster mainly consisted of sarcoidosis patients which was characterized with microbial community monopolized by Erythrobacteraceae family. Our results suggested the importance of considering the influence of oral microbiota in evaluating lung microbiota.

## Introduction

Granulomatosis with polyangiitis (GPA) and microscopic polyangiitis (MPA) are forms of antineutrophil cytoplasmic antibody (ANCA)-associated vasculitis (AAV), a systemic disease affecting multiple organs including the lungs and kidneys. The production of ANCA, playing a pathogenetic role in AAV, is thought to be initiated by exogenous or endogenous antigens including microbes, drugs, or dysregulated autoantigen expression^1^. Patients with GPA have a higher rate of *Staphylococcus aureus* carriage in their noses^2^ and bronchoalveolar lavage fluid (BALF)^3^.

Sarcoidosis is a granulomatous disorder affecting multiple organs, characterized by a non-caseating granuloma, the hallmark of sarcoidosis. The non-caseating granuloma is thought to be the result of immunological responses to antigenic triggers including spatial, seasonal, occupational, and infectious factors^4^. Numerous infectious agents have been suggested as possible etiologic agents of sarcoidosis, including mycobacteria and cutibacteria (formerly propionibacteria)^5^.

Contributions of mycobacteria to sarcoidosis have been suggested by studies of acid-fast cell wall-deficient forms of bacteria^6^ and a mycobacterial antigen, *Mycobacterium tuberculosis* catalase-peroxidase (mKatG)^7^. *Propionibacterium acnes* (*Cutibacterium acnes*) is also associated with sarcoidosis, as described in a study using lymph nodes of patients with sarcoidosis^8^. The identification of etiologic bacteria to sarcoidosis is challenging despite the above-mentioned findings, and novel concepts and techniques are required for this purpose.

The advent of new technologies using high-throughput sequencing has made it possible for us to evaluate and understand compositional differences in the microbiota of body sites between health and disease^9^. Because novel techniques for microbial identification are culture-independent, they have demonstrated diverse communities of microbes even in body sites including lung which are historically considered sterile in healthy status^10^. As for autoimmune diseases, patients with rheumatoid arthritis had less diversity and abundance of microbiota compared with healthy controls^11^.

We hypothesized that the difference of lung microbiota between AAV and sarcoidosis, both are diseases with lung involvement, would characterize each disease. In addition, we assumed that disease activity of AAV would associate with lung microbiota. Given these hypotheses, we herein evaluated the lung microbiota of AAV and sarcoidosis. We detected microbes that were reported to exist in the oral cavity also in our BALF samples in the analytic process and had a hypothesis that oral microbes might affect lung microbiota. However, we had no our own samples from the oral cavity nor data of oral microbiota. Therefore, we used data of oral microbiota from the Human Microbiome Project (HMP)^12^ to evaluate the effect of oral microbiota on lung microbiota.

## METHODS

### Patients and study criteria

We enrolled patients newly diagnosed with AAV or sarcoidosis between October 2014 and February 2018 at Nagasaki University Hospital and Sasebo Chuo Hospital. All patients with AAV were diagnosed based on the Chapel Hill Consensus Conference criteria^13^ and the European Medicines Agency algorithm^14^. The patients’ diagnoses included two types of AAV: GPA and MPA.

The diagnosis of sarcoidosis was histopathologically confirmed according to the consensus criteria of the American Thoracic Society/European Respiratory Society^15^. We excluded patients with tuberculosis and nontuberculous mycobacteria based on the results of the BALF culture and polymerase chain reaction (PCR). We also excluded patients with other diseases that could cause the same histopathological findings as sarcoidosis^16^.

We collected the demographic and clinical characteristics including organ involvement, and laboratory data at diagnosis. Smoking status was defined as “current smoker”, “former smoker”, and “never smoker.” Disease activities of AAV at diagnosis were assessed using Birmingham vasculitis activity score (BVAS)^17^. None of the patients had received corticosteroids or other immunosuppressive therapies at the time of BALF collection.

This study was performed in accordance with the Declaration of Helsinki and was approved by the Institutional Review Board of Nagasaki University Hospital (registration no.: 14122251) and Sasebo Chuo Hospital (registration no.: 2018-29). Informed consent for the use of their data was obtained from all of the patients.

### Bronchoalveolar lavage and cell preparation

BALF was collected with four instillations of sterile physiological saline (50 mL) through a flexible bronchoscope, and the fluid was immediately retrieved by gentle suction using a sterile syringe. The fluid was gently suctioned back to a bottle kept on ice. We used the fourth BALF withdrawal for the DNA extraction.

### DNA extraction

We collected pellets from BALF after centrifugation for 30 min at 13,000 *g* at 4 °C and the removal of supernatants. We stored pellets at −80 °C until processing. DNA extraction from the pellets was performed using a PowerBiofilm DNA Isolation Kit (MoBio Laboratories, Carlsbad, CA).

### Sequence analysis

High-throughput sequencing of bacterial 16S rRNA genes amplicon and phylogenetic tree construction from the FASTQ-format outputs were conducted by the Bioengineering Lab Co. (Kanagawa, Japan). Bacterial 16S rRNA gene amplicons encoding the V4 region (300 or 250 read length, paired-end protocol) were sequenced using a MiSeq Illumina sequencer (Illumina, San Diego, CA). The reads started with a 515F-806R primer pair^18^ and sequences were extracted as the 16S rRNA V4 region. The primer sequences and deeper than 251 bases from the primer sequences were trimmed using the FASTX-Toolkit (ver. 0.0.14) (http://hannonlab.cshl.edu/fastx_toolkit/).

For the quality filtering, the threshold of quality score and length were set at 20 and 40, respectively, and basecalls which did not fulfill these criteria were not adopted. Forward and reverse reads with the length of 250 and 230 base pairs, respectively, were merged using FLASH (ver. 1.2.11, http://www.cbcb.umd.edu/software/flash) with default parameters other than these lengths. The merged reads with lengths of 240–260 base pairs were extracted using SeqIO in biopython. Chimeric reads identified using USEARCH with the reference sequence of Greengenes 13_8 were removed. The remaining sequences were clustered into operational taxonomic units (OTUs) using a 97% similarity threshold (without any external reference sequence collection) with the Quantitative Insights into Microbial Ecology (QIIME)^19^ pipeline with the default parameters (this process was accessed through *pick_de_novo_otus.py* command^20^).

### Body-site specific OTU table from healthy population

We downloaded the Human Microbiome Project (HMP)^12^ dataset, that was generated from samples obtained from 5 body sites and 15 or 18 subsites (the difference of three depends on the subjects’ sex) of 242 healthy adult without evidence of disease^12^, from the HMP data analysis and coordinating center (DACC) (https://www.hmpdacc.org/hmp/) via the R package *HMP16SData* v.1.4.1^21^. The details on data generation are published as two articles^12,22^.

### Statistical analysis

The association between variables was assessed using Fisher’s exact test for categorical variables and the Mann-Whitney U test for quantitative variables.

The α-diversity was measured by the inverse Simpson index that is derived from the Simpson index^23^. The Simpson index is known to be more robust against variation in sampling effort^24^ than the Shannon index. This robustness is inherited to the inverse Simpson index because the inverse Simpson index is simply an inverse of the subtraction of the Simpson index from 1. The β-diversity was measured by the Morisita-Horn dissimilarity index from the perspective of robustness against variation in sampling effort^25^. The differences in the α-diversity indices between diseases was evaluated with the Mann-Whitney U statistic. The *p*-values for the Mann-Whitney U tests were calculated via permutation test^26,27^. Among the AAV patients, the linear relationships between the α-diversity index and BVAS were analyzed as regression coefficients for the α-diversity index on BVAS by the linear regression. Confidence intervals for the regression coefficients were constructed via bootstrap resampling^28^. The contour of two-dimensional probability densities was drawn based on a kernel density estimate with a Gaussian kernel with bandwidth selected by the “solve-the equation” estimator^29,30^.

From an interest of the microbial mouth-lung immigration^31^, we evaluated the α- and β-diversities in the lung microbiota after extracting a set of taxa of “inhabitant” in the oral cavity. We did not collect oral specimens, therefore we presumed that bacterial taxa of the oral cavity “inhabitant” were present in our subjects’ oral cavity. A set of taxa of “inhabitant” was determined based on the prevalence of each taxon among the HMP subjects, for each of the nine oral cavity and oropharynx subsites from which specimens were collected in the HMP; concretely, taxon with a frequency (read count > 0) of over 98% of the specimens obtained from a subsite (that was determined based on the infimum of a binomial probability with which the lower boundary of >0.95 of the 95% confidence interval by the Wilson’s score method^32^ was determined as a member of the “inhabitant” in the subsite. This procedure was performed using the ANCOM-II^33^. In addition, because we assumed that microbes of oral sites can be the source of noise in lung microbiota, we analyzed the lung microbiota after exclusion of oral microbes which can be noise. We defined taxa of the oral sites that over 5% of the HMP subjects had as the“vagrant.” An effect of selecting a specific set of taxa, which owed to extraction of “inhabitants” or exclusion of “vagrant”, on the differences in α-diversities between disease groups, were measured as a percentile rank of a Mann-Whitney’s U statistic from observed data on the empirical cumulative distribution function (ECDF) of the U statistics constructed from 2,000 sets of random drawn taxa. The respective numbers of the drawn taxa in the analysis was determined by the number of the taxa in the analysis. The random drawings were conducted without replacement. On the AAV patients, by switching the U statistic to the regression coefficients for α-diversity regressed on BVAS, the effect of selecting specific set of the taxa were evaluated on the ECDF constructed from the regression coefficients for the inverse Simpson index regressed on BVAS for random drawn 2,000 set of taxa.

The hierarchical clustering was conducted on the β-diversity by the complete linkage method. In the respective heatmaps, taxa were sorted with single linkage method on the Jaccard distances between taxa.

The effects of clinical factors on β-diversity were determined by a permutational multivariate analysis of variance (PERMANOVA)^34^. The PERMANOVA was conducted with complete cases for each dependent variable (there was a sample with missing values regarding the percentages of macrophages, lymphocytes, eosinophils, and neutrophils of BALF). All the reported p-values are descriptive^35^. All the statistical analyses were conducted under the R environment v. 3.6.0^36^ using relevant packages (*vegan* v. 2.5.5^37^*, metagenomeSeq* v.1.26.0^38^*, phyloseq* v. 1.28.0^39^*, coin* v. 1.3.0^40^, and *boot* ver. 1.3–22^41)^. Source codes used in the statistical analyses are available from https://github.com/mrmtshmp/microbiome_AAV.

## RESULTS

### Patient characteristics

**Figure 1:**
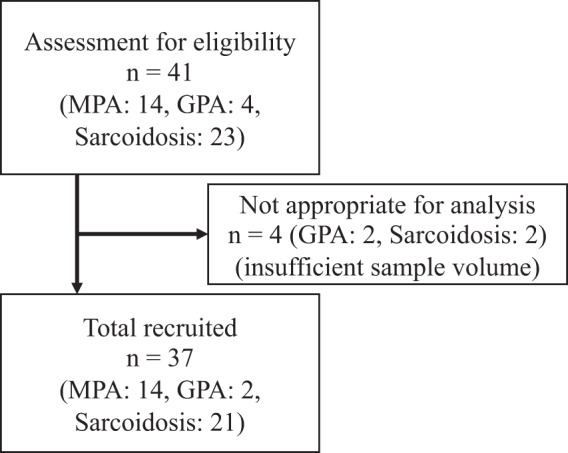
The flow diagram of inclusion.

Total 37 patients (16 AAV and 21 sarcoidosis) were recruited in this study (Fig. 1). Table 1 summarizes the demographic and clinical characteristics of the patients. The patients with AAV were older than the patients with sarcoidosis (median 78 yrs vs. 62 yrs, *p* = 0.0002). The median ages at diagnosis were reported to be 66.8 years old in GPA and 70.5 years old in MPA in the Japanese population^42^. Whereas the median age at diagnosis of sarcoidosis was reported to be 54 years old in the Japanese population^43^. Therefore, the older age at diagnosis of AAV compared with sarcoidosis in our patients was consistent with those in the nation-wide studies. Smoking status was not different between the disease groups. Thirteen patients were positive for myeloperoxidase (MPO)-ANCA. The median BVAS was 16 (interquartile range (IQR) : 8 to 19). All patients with AAV had lung involvement. Eighteen of the 21 patients with sarcoidosis had lung involvement. The patients with AAV had a lower recovery percentage of BALF compared to that of the patients with sarcoidosis. The patients with AAV had lower percentages of lymphocytes and higher percentages of neutrophils in BALF cells compared to the sarcoidosis group. Because BALF from patients with GPA with high disease activities had lower percentages of lymphocytes and higher percentages of neutrophils compared with those from patients with sarcoidosis^44^, our data were consistent with the previous report.

**Table 1:** Demographic and clinical characteristics of the patients with AAV and sarcoidosis.

	AAV, n = 16	Sarcoidosis, n = 21	p-value
Female, n (%)	11 (69)	15 (71)	1.00
Age, yrs, median (IQR)	78 (75–81)	62 (45–73)	0.0002
**Smoking:**			
Never smokers, n (%)	11 (69)	11 (52)	
Former smokers, n (%)	3 (19)	4 (19)	0.5234*
Current smokers, n (%)	2 (13)	6 (29)	
MPO-ANCA-positive, n (%)	13 (81)		
MPO-ANCA titer, U/mL (range)	43 (15–172)	—	
PR3-ANCA positive, n (%)	1 (6)		
PR3-ANCA titer, U/mL	34 (n = 1)	—	
GPA/MPA	2/14	—	
**AAV involvements, n (%):**			
Lung	16 (100)	—	
Kidney	7 (44)	—	
ENT	1 (6)	—	
Nerve	3 (19)	—	
Eye	1 (6)	—	
Joint	2 (13)	—	
BVAS, median (IQR)	16 (8–19)	—	
**Sarcoidosis involvements, n (%):**			
Lung	—	18 (86)	
Eye	—	11 (52)	
Lymph node	—	18 (86)	
Skin	—	5 (24)	
Heart	—	3 (14)	
Pancreas	—	1 (5)	
**Bronchoscopy:**			
Recovery percentage of BALF (%), median (IQR)	34 (31–44)	53 (48–62)	<0.0001
BAL fluid cell concentration (10^5^ cells/mL), median (IQR)	2.9 (2.0–6.0)	2.2 (1.9–3.5)	0.2102
Macrophages (%), median (IQR)	52 (46–61)	63 (48–83)	0.1352
Lymphocytes (%), median (IQR)	12 (6–28)	36 (14–50)	0.0052
Neutrophils (%), median (IQR)	22 (5–45)	1 (0–3)	<0.0001
Eosinophils (%), median (IQR)	0 (0–3)	0 (0–2)	0.5302

*Cochran-Armitage test among never smokers, former smokers, and current smokers. AAV: ANCA-associated vasculitis, ANCA: antineutrophil cytoplasmic antibody, BALF: bronchoalveolar lavage fluid, ENT: ear: nose: throat, IQR: interquartile range, MPO: myeloperoxidase, PR3: proteinase 3.

### α-diversity and β-diversity in lung-microbiota

The depth of sequencing was 14,986 reads per sample in median (IQR: 8,467 to 24,354) which passed the quality check explained in the section for Sequence analysis. There was no difference in depth of sequencing between the groups of subjects with AAV and sarcoidosis (median [IQR]: 16,786 [4,705 to 29,857] in AAV, 14,148 [7,608 to 24,354] in sarcoidosis, *p* = 0.57). The proportions of read counts from OTUs without taxonomic names in each subject’s sample were depicted as boxplots (Fig. S1A).

**Figure 2:**
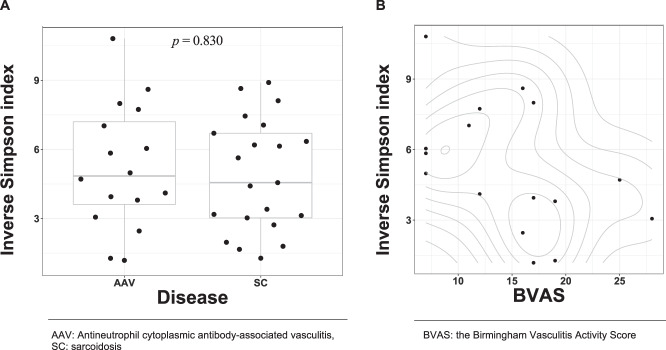
The association of the α-diversity with the diseases and BVAS. (**A**) The difference in the inverse Simpson index between diseases. The width of the dots’ distribution on each box plot was based on corresponding kernel-density estimation with Gaussian-kernel. Each box denotes corresponding interquartile range and each horizontal line in the middle of each box denotes the median of corresponding density estimates. (**B**) The bivariate relationship between the inverse Simpson index and BVAS. The contour lines show the two-dimensional probability density obtained from a kernel density estimate.

The difference in the inverse Simpson index between disease groups (AAV and sarcoidosis groups) was not observed (*p* = 0.830, Fig. 2A). We did not observe the association between the recovery percentage of BALF and the inverse Simpson index (Fig. S2). The observed *p*-values for differences in the inverse Simpson index between sexes or the smoking status were as follows; *p* = 0.273 for female vs. male subjects, *p* = 0.778 for current vs. never smokers, *p* = 0.575 for former vs. never smokers, *p* = 0.817 for current vs. former smokers. (Figs. S3 and S4).

A linear relationship between the inverse Simpson index and BVAS was shown in a scatter plot (Fig. 2B). The regression coefficient for the inverse Simpson index on BVAS was −0.206 (95%CI: −0.373 to −0.085).

**Figure 3:**
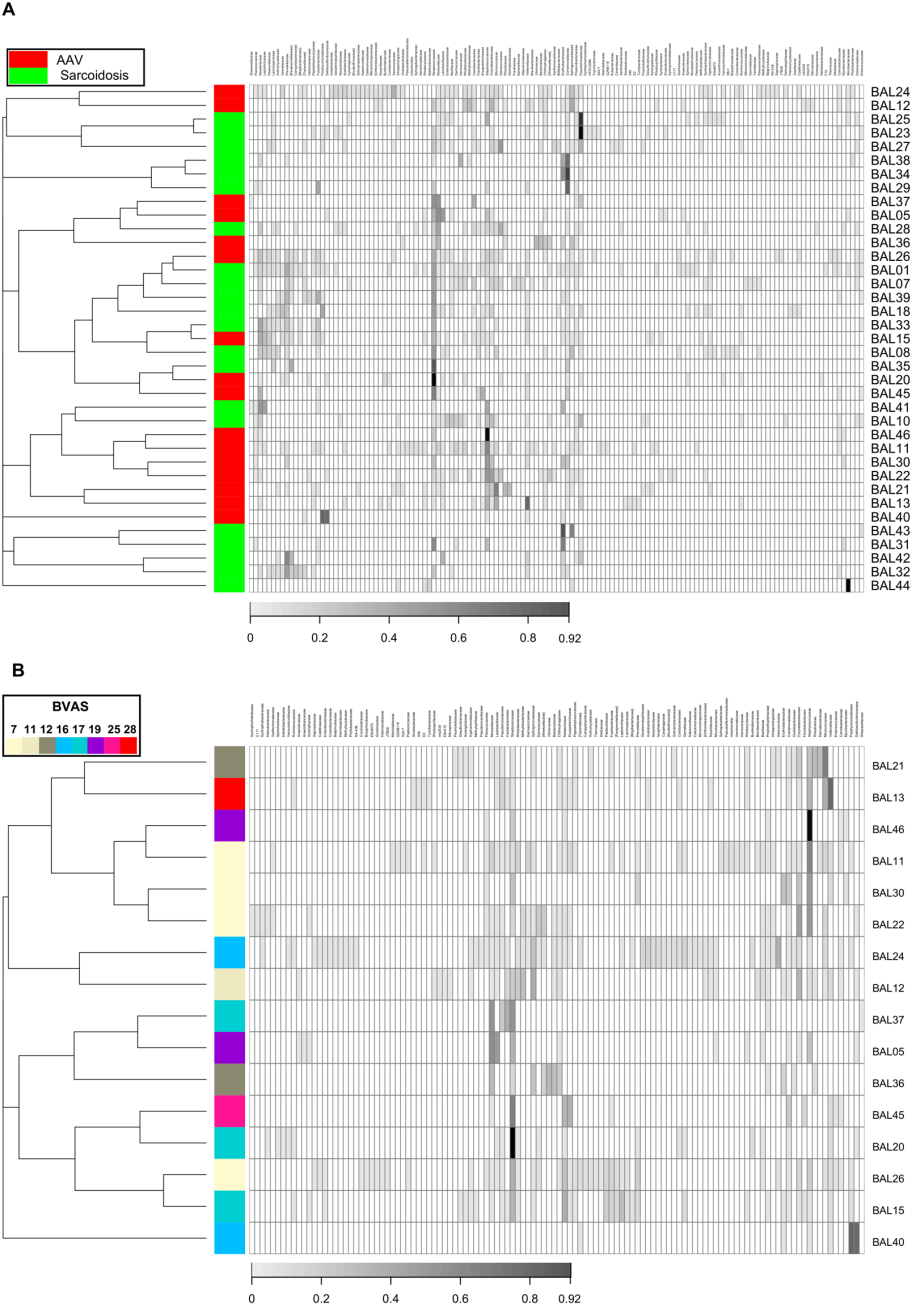
The microbiota composition and dendrogram of the subjects. The grayscales in heatmaps represent the relative abundance of each taxon. (**A**) The red and green tiles on the first column in the heatmaps denote respective disease groups (AAV and sarcoidosis) to which corresponding subjects belonged. (**B**) The colour of each tile on the first column in the heatmap indicate BVAS in corresponding subject. For both of (**A**) and (**B**), the dendrograms in the left side of the heatmaps show results from hierarchical clustering with the complete linkage method on the Morisita-Horn dissimilarity index. The taxa were sorted by hierarchical clustering with single linkage method on the Jaccard distance between rows.

The observed *p*-value for the effect of the disease groups on the Morisita-Horn dissimilarity index at the family rank was 0.0331 from the PERMANOVA. The dendrogram on the heatmap (Figs. 3A and S6) showed that small clusters consisted only of patients with each disease (namely, clusters with patient’s IDs of {12, 24}, {27, 23, 25}, {29, 34, 38}, {10, 41}, {22, 30, 11, 46}, {13, 21} and {32, 42, 31, 43}). However, we observed neither large clusters nor evident patterns in the heatmap that were shared with the patients with each disease despite the small *p*-value was observed from the PERMANOVA.

We evaluated the association between BVAS and the Morisita-Horn dissimilarity index (Fig. 3B, *p* = 0.3588, PERMANOVA). The effect of other physiological factors (age, sex, smoking status, percentage of macrophages, lymphocytes, eosinophils, and neutrophils in BALF) on the Morisita-Horn dissimilarity index were tabulated in the Table 2. We did not observe any patterns of clusters associated with BVAS in the heatmap (Figs. 3B and S6).

**Table 2:** The p-values of physiological factors on β-diversity indices.

Taxonomic rank	AAV/Sarcoidosis n = 37	BVAS n = 16	Age n = 37	Sex n = 37	Smoking status Never + Former vs. Curr. n = 37	Smoking status Never vs. Curr. + Former n = 37	Macrophage n = 36	Lymphocyte n = 36	Neutrophil n = 36	Eosinophil n = 36
Family	0.0331	0.3588	0.9241	0.9793	0.7986	0.9221	0.2027	0.1364	0.0599	0.4697
Genus	0.0792	0.3436	0.9103	0.9623	0.7866	0.8253	0.2014	0.0618	0.0935	0.4943

We detected Prevotellaceae, Veillonellaceae, and Streptococcaceae, which have been detected in BALF of healthy individuals in previous researches^31,45–47^ in our BALF samples (Fig. 3A). Therefore, we aimed to evaluate the contributions of oral microbes on the lung microbiota.

### Lung-microbiota diversity analysis focusing on oral inhabitant taxa

As we did not collect oral specimen in our study, we aimed to determine the oral taxa commonly shared in healthy individuals by using the HMP dataset. We extracted taxa that were detected with 0.98 or higher frequency in each oral subsite and determined them as subsite’s inhabitant taxa. Furthermore, we validated the stability of the selected taxa by seeing how many numbers of taxa shared by 98% of subjects were retained as the percentage of subjects was increased up to 99.5% (Fig. S1B). We found that “hard palate” as an oral subsite had a stable number of taxa because 11 taxa at family rank of “hard palate inhabitant” shared by 98% of subjects were retained by 99% of subjects while other body sites’ numbers of taxa shrank. Therefore, we presumed that the 11 taxa of “hard palate inhabitant” were shared commonly in our subjects. The identified 11 taxa as “hard palate inhabitant” were as follows (alphabetical order);  Actinomycetaceae, Carnobacteriaceae, Fusobacteriaceae, Gemellaceae, Lachnospiraceae, Neisseriaceae, Pasteurellaceae, Porphyromonadaceae, Prevotellaceae, Streptococcaceae, and Veillonellaceae.

**Figure 4:**
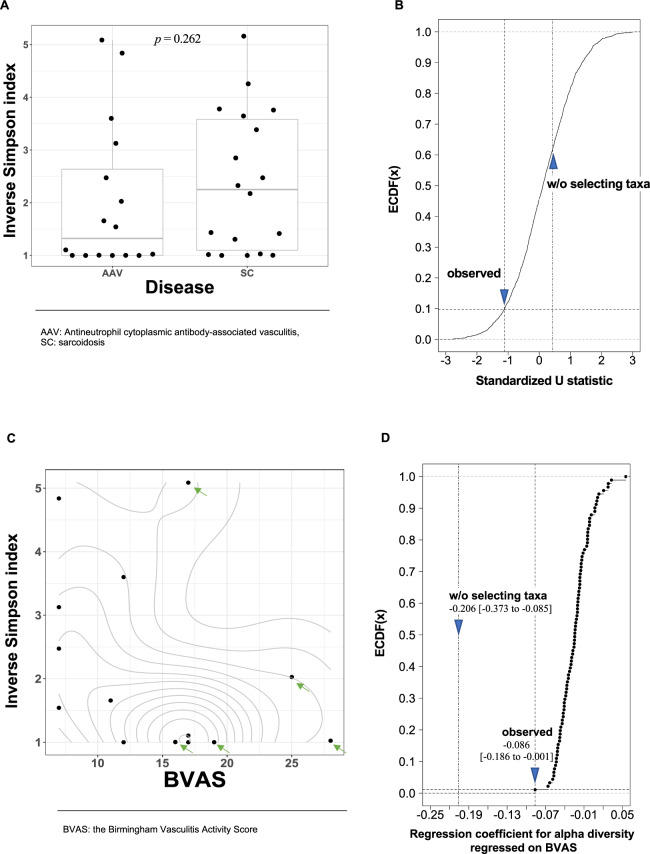
The association of the α-diversity in the set of taxa of “hard palate inhabitant” with the diseases and BVAS. (**A**) The difference in the inverse Simpson index between diseases. The width of the dots’ distribution on each box plot was based on corresponding kernel-density estimation with Gaussian-kernel. Each box denotes corresponding interquartile range and each horizontal line in the middle of each box denotes the median of corresponding density estimates. (**Β**) The effect of selecting the 11 taxa of “hard palate inhabitant” contrasted with random drawning of 11 taxa on the association between the α-diversity and the diseases. The standardized U statistics were calculated for the difference in the inverse Simpson index between diseases. The two blue triangles depict the standardized U statistics calculated from the inverse Simpson index for the taxa of “hard palate inhabitant” (“**observed”**) and all of the detected taxa (“**w/o selecting taxa”**), respectively. (**C**) The bivariate relationship between the inverse Simpson index and BVAS. The contour lines show the two-dimensional probability density obtained from a kernel density estimate. Each of the green arrows indicates a male subject. (**D**) The effect of selecting the 11 taxa of “hard palate inhabitant” contrasted with random drawning of 11 taxa on the association between the α-diversity and BVAS. The regression coefficient was for the inverse Simpson index regressed on BVAS. The two blue triangles depict the regression coefficient calculated from the inverse Simpson index for the taxa of “hard palate inhabitant” (“**observed”**) and all of the detected taxa (“**w/o selecting taxa”**), respectively. The figures under the blue triangles are regression coefficients and their 95% confidence intervals.

We observed a clearer difference in α-diversity between the disease groups on the 11 taxa of “hard palate inhabitant” (Fig. 4A, *p* = 0.262, Wilcoxon rank sum test for the inverse Simpson index) than that on all the detected taxa. The percentile rank of the U statistic was 9.1% on the ECDF constructed from U statistics for 2,000 random drawing of 11 taxa (Fig. 4B). To confirm that observed effect of selecting the set of taxa of “hard palate inhabitant” was not a result from the association between sexes and α-diversity, we reanalyzed after stratification by the sexes. As results, we observed percentile ranks those were closer to 50.0% than analysis without the stratification (17.6% for female subjects and 26.1% for male subjects). Those increment in the percentile ranks, especially in the male subjects, suggested that the observed relationship was partly evoked from those between sexes and the α-diversity. The differences in the inverse Simpson index among smoking status were made clearer, but not sexes, when limiting to “hard palate inhabitant” (Figs. 3S and 4S).

The regression coefficient for the inverse Simpson index regressed on BVAS was estimated −0.086 (95%CI: −0.186 to −0.001) when limiting the included taxa to “hard palate inhabitant”. The absolute value of the regression coefficient became smaller compared with that estimated from all the detected taxa (−0.206 (95%CI: −0.373 to −0.085)) because of the decreased α-diversity by downsizing of the number of taxa from 144 to 11. While the regression coefficient for the inverse Simpson index shrank after limiting to “hard palate inhabitant”, the percentile rank was 1.1% on the ECDF. In the distribution of BVAS, we observed separation between sexes (green arrows in Fig. 4C depicted the plots of male subjects). Because we obtained the percentile rank of 0.4% for the female subjects and also 0.4% for the male subjects on the ECDF when stratifying subjects by sexes, we concluded the observed effect was not resulted from the separation of BVAS between sexes. The regression coefficients were −0.185 (95%CI: −0.321 to −0.076) for female subjects and −0.109 (95%CI: −0.511 to 0.133) for male subjects.

**Figure 5:**
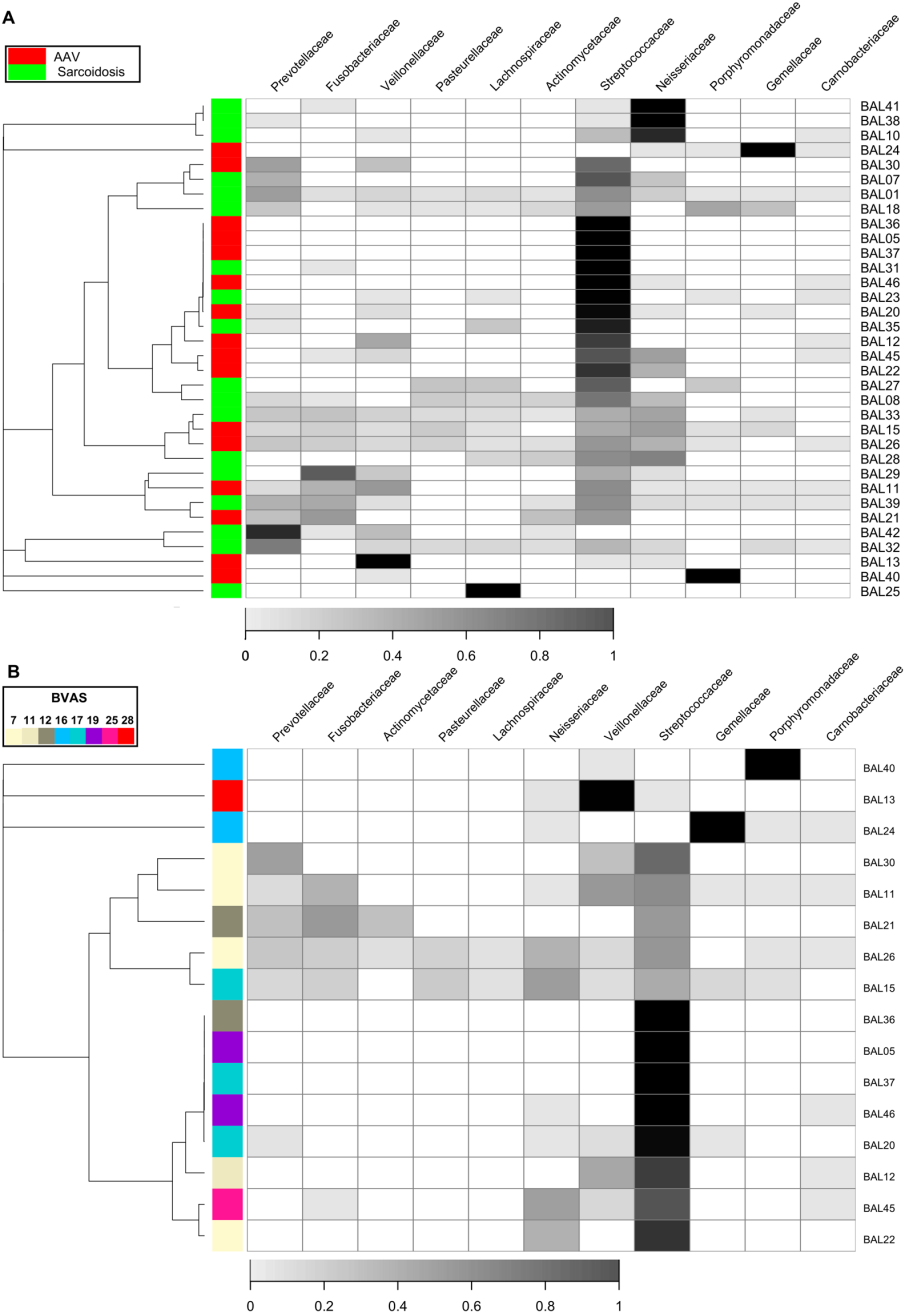
The microbiota composition and dendrogram of the subjects focusing on the taxa of “hard palate inhabitant”. The grayscales in heatmaps represent the relative abundance of each taxon. (**A**) The red and green tiles on the first column in the heatmaps denote respective disease groups (AAV and sarcoidosis) to which corresponding subjects belonged. (**B**) The colour of each tile on the first column in the heatmap indicate BVAS in corresponding subject. For both of (**A**) and (**B**), the dendrograms in the left side of the heatmaps show results from hierarchical clustering with the complete linkage method on the Morisita-Horn dissimilarity index. The taxa were sorted by hierarchical clustering with single linkage method on the Jaccard distance between rows.

The effect of disease groups on the Morisita-Horn dissimilarity index (*p* = 0.1410, PERMANOVA) was diminished when limiting to “hard palate inhabitant” compared with that from all the detected taxa. We observed no evident patterns in the heatmap that were shared with the patients with each disease (Figs. 5A and S6). We observed the diminished effect of BVAS on the β-diversity (*p* = 0.5907, PERMANOVA for Morisita-Horn dissimilarity index) when limiting to “hard palate inhabitant” compared with that from all the detected taxa. We did not observe any patterns of clusters associated with BVAS in the heatmap (Figs. 5B and S6).

Results from analyses on taxa of inhabitant in other oral subsites (that include saliva, attached keratinized gingiva, buccal mucosa, hard palate, palatine tonsils, sub and supra gingival plaque, throat and tongue dorsum) are shown in supplementary data (Figs. S7–S10).

### Lung-microbiota diversity analysis after noise reduction

To avoid the influence of oral microbes on lung microbiota, we evaluated associations between the diseases and the lung microbiota after excluding the taxa of “vagrant”. After the exclusion, the number of taxa in the OTU table was 48 at family rank.

**Figure 6:**
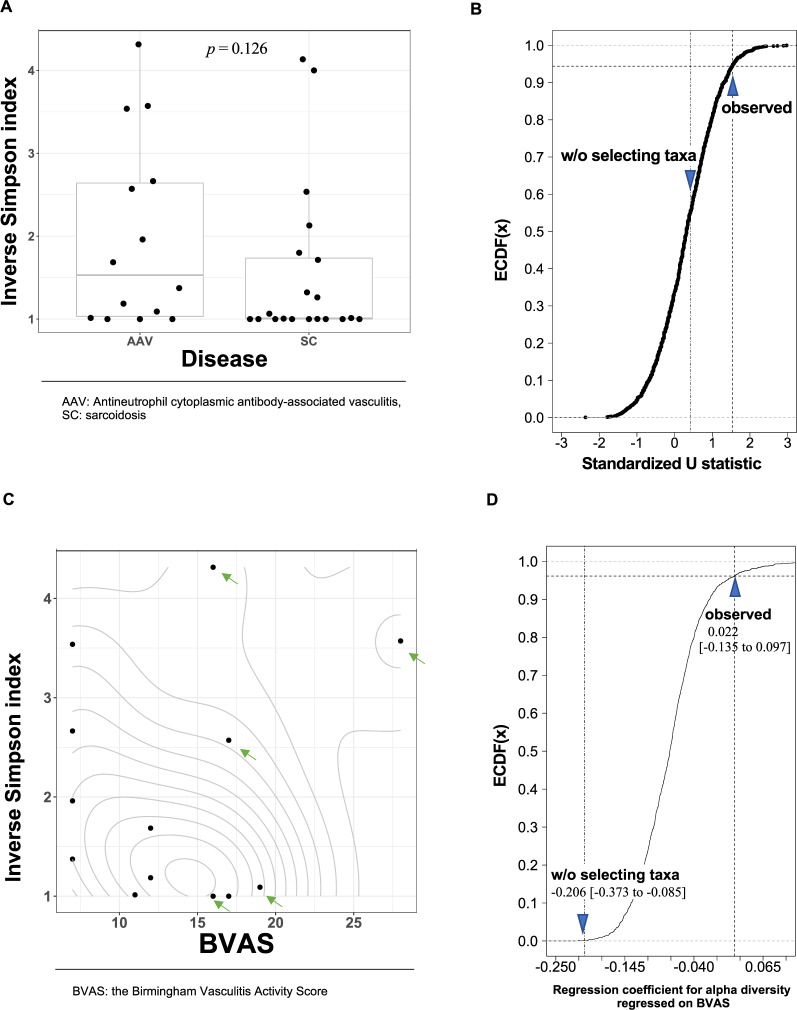
The association of the α-diversity in the set of taxa from which the set of taxa of “vagrant” was excluded, with the diseases and BVAS. (**A**) The difference in the inverse Simpson index between diseases. The width of the dots’ distribution on each box plot was based on corresponding kernel-density estimation with Gaussian-kernel. Each box denotes interquartile range and each horizontal line in the middle of each box denotes the median of corresponding density estimate. (**Β**) The effect of selecting the 48 taxa after excluding the taxa of “vagrant” contrasted with random drawning of 48 taxa on the association between the α-diversity and the diseases. The standardized U statistics were calculated for the difference in the inverse Simpson index between diseases. The two blue triangles depict the standardized U statistics calculated from the inverse Simpson index for the set of taxa from which the set of taxa of “vagrant” was excluded (“**observed”**), and all of the detected taxa (“**w/o selecting taxa”**), respectively. (**C**) The bivariate relationship between the inverse Simpson index and BVAS. The contour lines show the two-dimensional probability density obtained from a kernel density estimate. Each of the green arrows indicates a male subject. (**D**) The effect of selecting the 48 taxa after excluding the taxa of “vagrant” contrasted with random drawning of 48 taxa on the association between the α-diversity and BVAS. The regression coefficient was for the inverse Simpson index regressed on BVAS. The two blue triangles depict the regression coefficient calculated from the inverse Simpson index for the 48 taxa after excluding the taxa of “vagrant” (“**observed”**) and all of the detected taxa (“**w/o selecting taxa”**), respectively. The figures under the blue triangles are regression coefficients and their 95% confidence intervals.

The observed *p*-value for the difference in the inverse Simpson index between diseases on the 48 taxa was 0.126 (Fig. 6A), which was smaller than both of the observed *p*-value from that on all the detected 144 taxa (*p* = 0.830) and that on the 11 taxa of “hard palate inhabitant” (*p* = 0.262). In addition, in the inverse Simpson index between diseases, we observed oppositely directed difference, compared with the result from the analysis on the taxa of “hard palate inhabitant”. The percentile rank of the U statistic was 94.2% on the ECDF (Fig. 6B). To confirm that this effect was not a result from the association between sexes and α-diversity, we reanalyzed after stratification by the sexes. As results, we observed that percentile ranks were still high but closer to 50.0% than that obtained from analysis without stratification (the percentile ranks of 90.6% for female subjects and 85.6% for male subjects). Consequently, we concluded that the observed effect was mainly of exclusion of the taxa of “vagrant” though was partly evoked from those between sexes and the α-diversity.

The regression coefficient for the inverse Simpson index regressed on BVAS was estimated 0.022 (95%CI: −0.135 to 0.097) when excluding “vagrant” (Fig. 6C). The percentile rank of the observed regression coefficient was 97.0% on the ECDF (Fig. 6D). We observed separation in BVAS between sexes (green arrows in Fig. 6C depicted the plots of male subjects), therefore we again stratified the subjects by the sex and reanalyzed on the association between these two variables. The analysis resulted to reveal that the effect of the exclusion of the “vagrant” on the association between BVAS and the inverse Simpson index did not differ from those of drawing randomly 48 taxa in the female subjects (the percentile rank was 59.4%). Regarding the male subjects, the percentile rank was remained high (94.5%). We show the effects of the sex and smoking status on the inverse Simpson index in Fig. S5.

**Figure 7:**
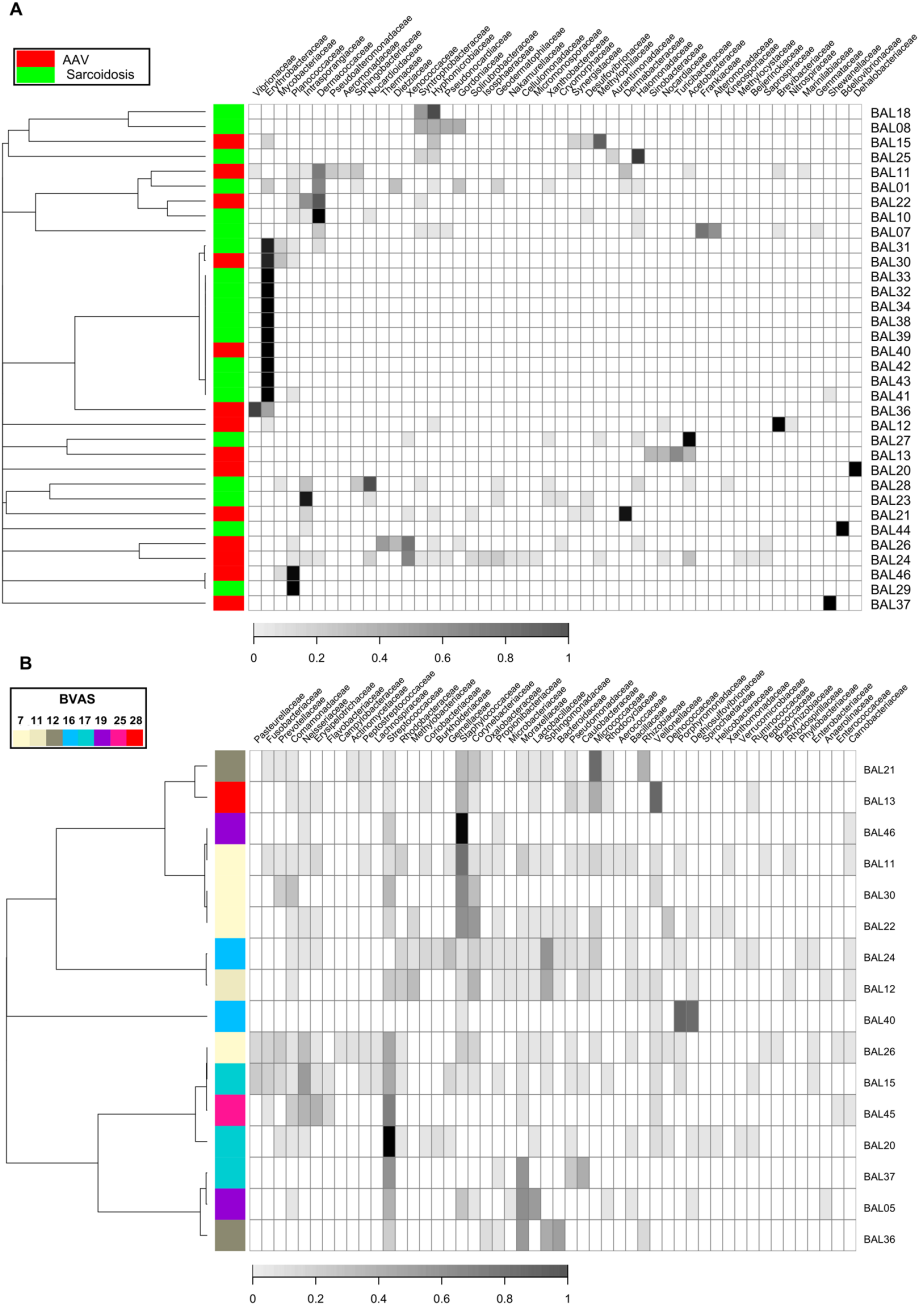
The microbiota composition and dendrogram of the subjects obtained from an analysis from which the set of taxa of “vagrant” was excluded. The grayscales in heatmaps represent the relative abundance of each taxon. (**A**) The red and green tiles on the first column in the heatmaps denote respective disease groups (AAV and sarcoidosis) to which corresponding subjects belonged. (**B**) The colour of each tile on the first column in the heatmap indicate BVAS in corresponding subject. For both of (**A**) and (**B**), the dendrograms in the left side of the heatmaps show results from hierarchical clustering with the complete linkage method on the Morisita-Horn dissimilarity index. The taxa were sorted by hierarchical clustering with single linkage method on the Jaccard distance between rows.

Through the β-diversity analyses with the OTU table after exclusion of the taxa of “vagrant”, we observed the diminished effect of disease groups on the microbiota composition dissimilarity between subjects (*p* = 0.1514, PERMANOVA for Morisita-Horn dissimilarity index) compared with that on all the detected taxa. The clustering analysis revealed a large cluster consisted of mainly subjects with sarcoidosis (9 of the 11 members) that was characterized by the monopoly in the microbiota composition by the Erythrobacteraceae family (Figs. 7A and S6). Regarding the effect of BVAS on the β-diversity, observed *p*-value was smaller (*p* = 0.2185, PERMANOVA for Morisita-Horn dissimilarity index) than that from observation on all the detected taxa. We did not observe any patterns of clusters associated with BVAS in the heatmap (Figs. 7B and S6).

## DISCUSSION

We have found associations between lung microbiota and two diseases as follows; first, the α-diversity negatively related with BVAS in the AAV group. Secondly, although no differences in the α-diversity was detected between AAV and sarcoidosis when evaluating all of the detected taxa, we found clearer differences between them when limiting the included taxa to “hard palate inhabitant” or excluding “vagrant”. Thirdly, we found the effect of the disease groups on the β-diversity with small *p*-value. Fourthly, although we found no distinctive patterns of clusters by all the detected taxa, the cluster characterized by the existence of Erythrobacteraceae family was consistent with the sarcoidosis group when excluding the “vagrant.”

Our result that the α-diversity negatively related with BVAS is consistent with the previous study using nasal swabs in GPA, which showed reduced α-diversity in patients with active disease (BVAS ≥ 1) compared with those in remission (BVAS = 0) while it was not significant^48^. The relationships between the disease severity and α-diversity have been reported in chronic obstructive pulmonary disease (COPD). The reduced α-diversity of the microbiota were observed in severe COPD when compared with mild COPD in BALF^49^ and sputum^50,51^. Although the question is whether the reduced α-diversity is the direct cause of increased disease activity (local or systemic inflammation) or merely its consequence, it remains unanswered by our result. Interaction with each other may form the vicious circle that causes persistent inflammation and dysbiosis in lung.

Microbes are shared between upper and lower respiratory tracts in healthy individuals^31,45–47^. We suggested the influence of migration of oral microbes into lung should be taken in consideration when evaluating lung microbiota even in patients with diseases. The difference in α-diversity between two diseases depended on the selection of taxa which limited to “hard palate inhabitant” or excluded “vagrant.” Although we cannot confirm which selection of taxa appropriately reflects the difference between two diseases, opposite effects of selecting taxa to the difference of α-diversity between two diseases may suggest importance of oral microbes or microbes in lung that do not exist in oral cavity.

Whether AAV or sarcoidosis themselves associated with the detection of taxa of oral microbes is another question. We had no patients who had obvious dysphagia, aspiration, or other manifestations that cause the migration of oral microbes into lung. In addition, dysphagia is a rare manifestation in both AAV^52^ and sarcoidosis^53^. Whereas, it is suggested that gastroesophageal reflux disease (GERD)-associated microaspiration may lead to the progression of idiopathic pulmonary fibrosis (IPF) and IPF may increase intrathoracic pressure, which can aggravate GERD vice versa^54^. GERD is a common problem in sarcoidosis^55^, but not in AAV^56^. Therefore, diseases themselves, especially sarcoidosis, may have had impacts on the detection of taxa of oral microbes in our study.

Limiting taxa based on oral taxa led to the intriguing finding that a cluster with Erythrobacteraceae family was consistent with the part of sarcoidosis group when excluding “vagrant.” Members of Erythrobacteraceae family are Gram-negative, aerobic, rod-shaped or pleomorphic coccoid bacteria^57^. They are isolated from wild rice, cold-seep sediment, desert sand, tepid water, seawater, tidal flats, marine sediment, and marine invertebrates. No information on pathogenicity of Erythrobacteraceae for human is available.

We found the effect of the disease groups on the β-diversity with small *p*-value while other physiological factors did not have. For example, smoking status has been reported to have significant impacts on β-diversity in lung microbiota^58^, but, it did not have the effect with small p-value in our study.

Several microbes have been reported to associate with AAV or sarcoidosis. As for AAV, a study using quantitative culture experiments reported that *Staphylococcus aureus* in BALF was particularly associated with patients with GPA compared with patients with idiopathic pulmonary fibrosis (IPF)^3^. One study using nasal swabs reported decreased *Propionibacterium acnes* and *Staphylococcus epidermidis* in patients with GPA when compared with healthy controls^59^. Another study using nasal swabs reported increased relative abundance of *Planococcaceae* family and decreased *Moraxellaceae, Tissierellaceae, Staphylococcaceae*, and *Propionibacteriaceae* families in GPA when compared with healthy controls^48^. A study with culture experiments with nasal swabs reported *Staphylococcus pseudintermedius* in patients with GPA^60^. As for sarcoidosis, in addition to *Mycobacterium*^7^ and *Propionibacterium acnes*^8^, *Atopobium* and *Fusobacterium* have been reported as candidates for sarcoidosis-associated microbiota in BALF^61^. However, we could not find any differences in the relative abundance of taxa at family rank corresponding to these taxa between AAV and sarcoidosis in the present study. A research including BALF samples from 16 patients with sarcoidosis and 12 healthy controls also showed no significant microbial differences between two groups^62^.

We enumerate 4 probable causes of theses inconsistencies of results between ours and previous researches. First, because previous studies of these bacteria used the nasal swabs^48,59,60^, nasal carriage^2^, blood samples^6^, or lymph nodes^8,63^, these results may be attributable to the differences of specimens. Second, we compared AAV with sarcoidosis although one study compared GPA with IPF^3^ and others compared AAV or sarcoidosis with healthy controls. Third, because the 16S rRNA gene sequencing approach is reported to be not sensitive in identifying nontuberculous mycobacteria among airway samples^64^, our attempt to detect mycobacteria using 16S rRNA may be inappropriate. Finally, this study predominantly included MPA, not GPA.

This study has some limitations. First, we could not evaluate BALF specimens of healthy individuals, and we thus could not identify bacteria that are present in both AAV and sarcoidosis but not in healthy subjects. In addition, the absence of data from healthy persons may have affected the interpretation of α-diversity and β-diversity regarding AAV and sarcoidosis. Second, we could not assess the effects of GPA or MPA and MPO-ANCA or PR3-ANCA, because most of the patients with AAV were MPA. Further research is necessary to address the question of whether distinct dysbiosis exists among types of AAV. Third, we could not evaluate the contamination. In samples with very low amounts of microbial biomass including BALF, many of the true signals are masked by contaminating DNA^65^. The assessment of processes of bronchoscope, extraction of DNA, amplification and library preparation might have been helpful to exclude contamination. Fourth, we did not evaluate the background microbial taxa. The significant intrusion of the background microbiota through the bronchoscopic procedure and the kit may obscure results as suggested^66^. Fifth, although we used the fourth BALF withdrawal for the DNA extraction, we had no data regarding the microbial differences between the first to third BALF and the fourth BALF. Sixth, the present study was done in a small number of patients to assess lung microbiota from two hospitals in a regional part of Japan. Predominant patients with AAV are MPA and MPO-ANCA positive patients, not GPA nor PR3-ANCA positive patients, which is quite different from those of the US and European countries. Our results may not apply to patients with AAV and sarcoidosis in other regions. Lastly, we used data of oral microbiota from the HMP to select “hard palate inhabitant” and exclude “vagrant.” Because the subjects of HMP are completely different from our subjects in terms of many physiological factors including age and race. These large differences may hamper the evaluation of our data.

In conclusion, α-diversity between AAV and sarcoidosis did not differ, but the observed *p*-value for the effect of the disease groups on the β-diversity was small. The α-diversity negatively related with BVAS in the AAV group. Limiting taxa to oral taxa or excluding oral taxa made the difference in α-diversity between AAV and sarcoidosis clearer. By excluding oral taxa, we found a cluster with Erythrobacteraceae family which mainly consisted of sarcoidosis. Our results suggested the importance of considering the influence of oral microbiota in evaluating lung microbiota.

## Supplementary information

Supplementary materials.

## Data Availability

Data are available from DDBJ Sequence Read Archive (accession number: PRJDB8270). Data are accessed from the following URLS: (http://trace.ddbj.nig.ac.jp/BPSearch/bioproject?acc=PRJDB8270) (https://www.ncbi.nlm.nih.gov/bioproject/?term=PRJDB8270).
