# Supplementary material for: Comparison of lung microbiota between antineutrophil cytoplasmic antibody-associated vasculitis and sarcoidosis
Source: Sci Rep. 2020 Jun 11;10:9466. doi: 10.1038/s41598-020-66178-4 (PMC7289840; doi:10.1038/s41598-020-66178-4)
Supplement: Supplementary file 1 — Supplementary materials. [file 41598_2020_66178_MOESM1_ESM.pdf]

Supplemental material

Comparison of lung microbiota between antineutrophil cytoplasmic antibody-associated vasculitis and sarcoidosis

Shoichi Fukui MD, PhD[1,2,*], Shimpei Morimoto PhD[3,*], Kunihiro Ichinose MD, PhD[1,**], Shota Nakashima MD, PhD[4], Hiroshi Ishimoto MD, PhD[4], Atsuko Hara MD, PhD[4], Tomoyuki Kakugawa MD, PhD[4], Noriho Sakamoto MD, PhD[4], Yoshika Tsuji MD[5], Toshiyuki Aramaki MD, PhD[5], Tomohiro Koga MD, PhD[1,6], Shin-ya Kawashiri MD, PhD[1,2], Naoki Iwamoto MD, PhD[1], Mami Tamai MD, PhD[1], Hideki Nakamura MD, PhD[1], Tomoki Origuchi MD, PhD[1,7], Yukitaka Ueki MD, PhD[5], Shino Suzuki PhD[8], Hiroshi Mukae MD, PhD[4], Atsushi Kawakami MD, PhD[1]

[1]Department of Immunology and Rheumatology, [2]Department of Community Medicine, [3]Innovation Platform & Office for Precision Medicine, [4]Department of Respiratory Medicine, [6]Center for Bioinformatics and Molecular Medicine, and [7]Department of Rehabilitation Sciences, Nagasaki University Graduate School of Biomedical Sciences, Nagasaki, Japan
[5]Rheumatic and Collagen Disease Center, Sasebo Chuo Hospital, Sasebo, Japan
[8]Kochi Institute for Core Sample Research, X-star, Japan Agency for Marine-Earth Science and Technology (JAMSTEC), Nankoku, Japan

*These authors contributed this work equally.

****Correspondence to:** Dr. Kunihiro Ichinose, Department of Immunology and Rheumatology, Nagasaki University Graduate School of Biomedical Sciences, 1-7-1 Sakamoto, Nagasaki 852-8501, Japan. Tel.: +81-095-819-7262; Fax: +81-095-849-7270

Email: kichinos@nagasaki-u.ac.jp
Key words: ANCA-associated vasculitis, bronchoalveolar lavage, lung, microbiota, sarcoidosis

Running head: Lung microbiota in AAV and sarcoidosis

Figure S1

A

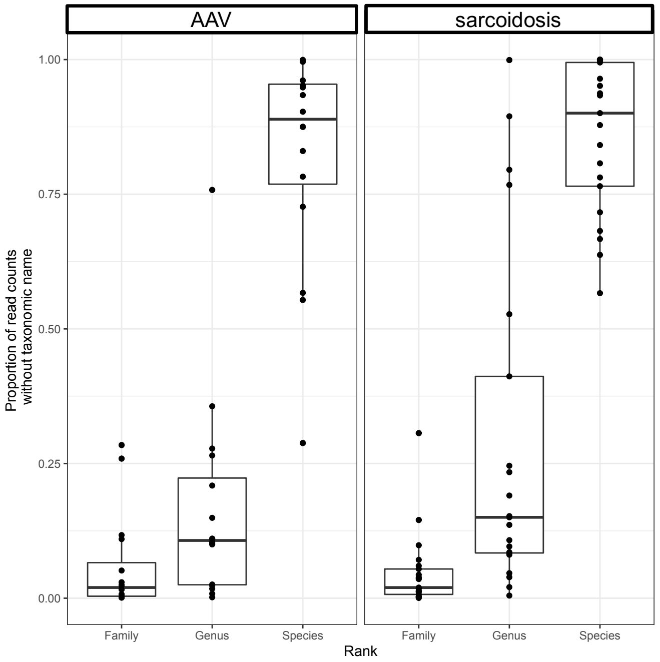

B

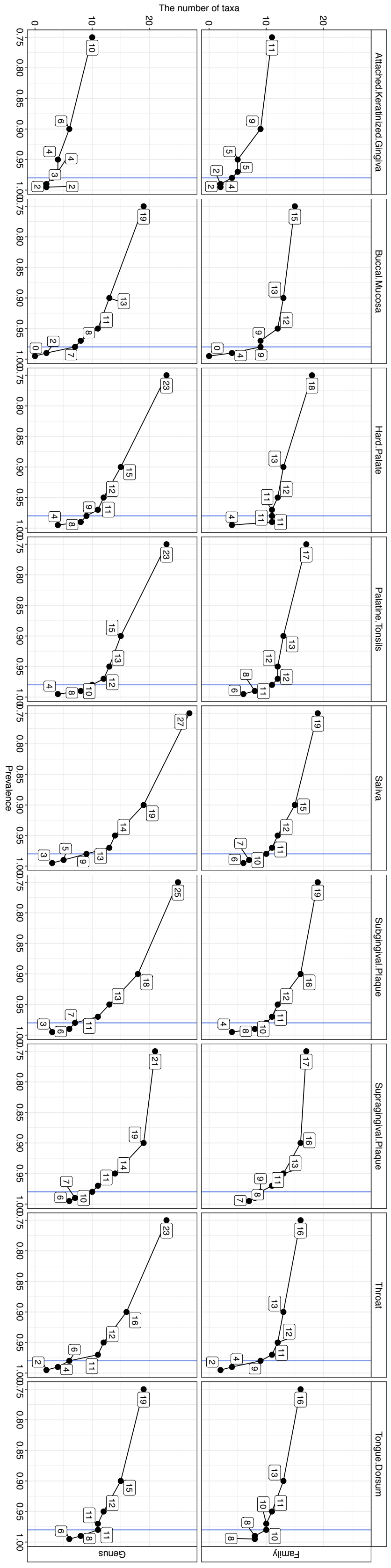

(A) The proportion of read counts from OTUs without the taxonomic name. The distribution of the proportions resembled to each other disease group at family rank [median (IQR): 2.0% (0.4 to 6.7%) in AAV and 2.0%(0.7 to 5.4%) in sarcoidosis].At genus rank, we observed difference in the distribution between disease groups (median [IQR]: 10.7% [2.5 to 22.3%] in AAV and 15.0% [8.4 to 41.2%] in sarcoidosis). At species rank, the proportions were > 75% in the three fourth subjects of each disease group.

(B) The number of taxa with the prevalence of 75, 90, 95, 97, 98, 99 and 99.5% in the HMP subjects. Each panel corresponds to each oral subsite in the HMP dataset. In the panels in the upper and lower row, the taxa were counted at family and genus rank, respectively.

Figure S2

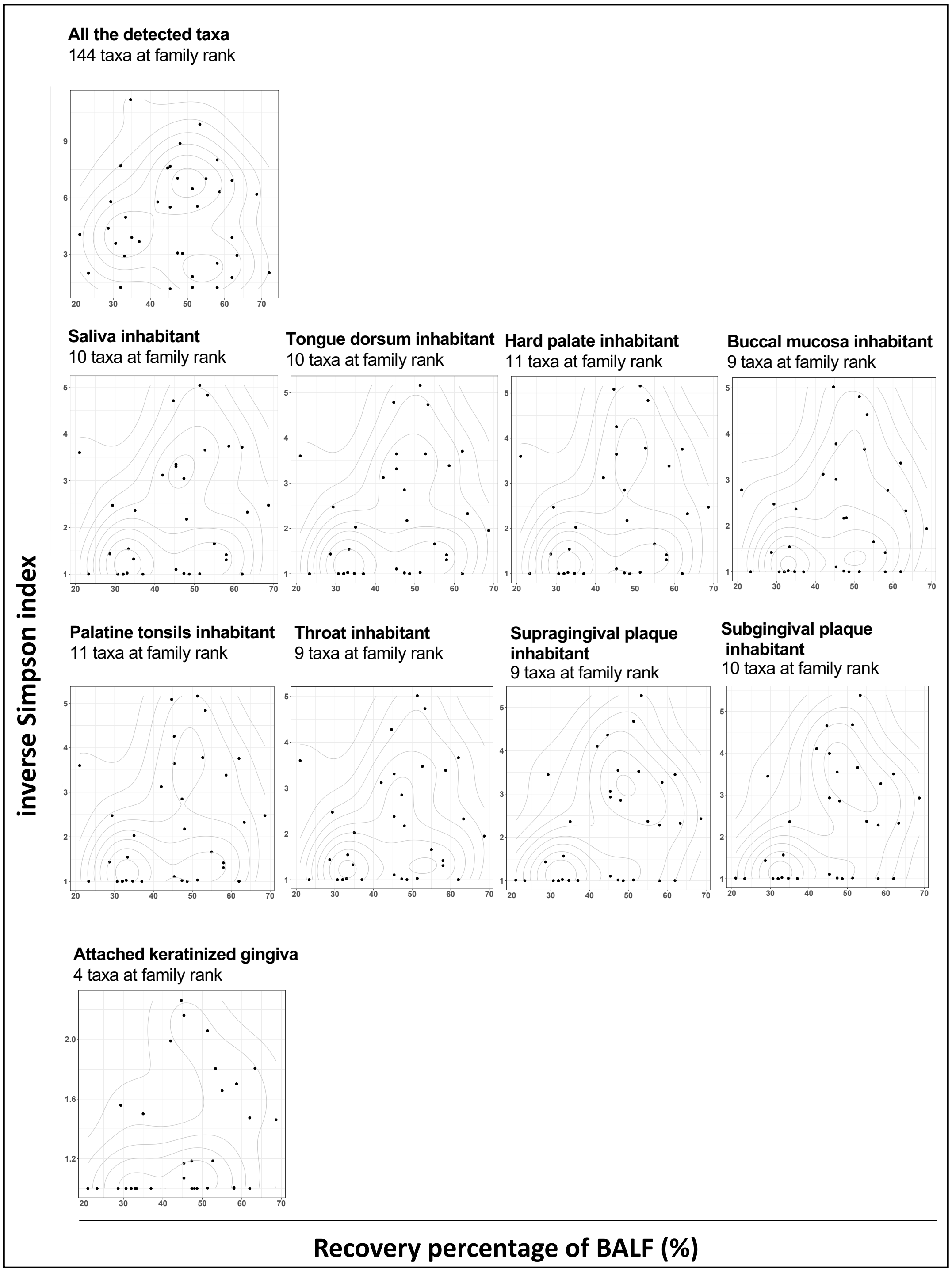

Figure S2. The bivariate relationship between the inverse Simpson index and the recovery percentage of BALF (%) evaluated for each set of taxa of inhabitant in the respective oral subsites. The contour lines are two-dimensional probability densities drawn based on corresponding kernel density estimate.

Figure S3

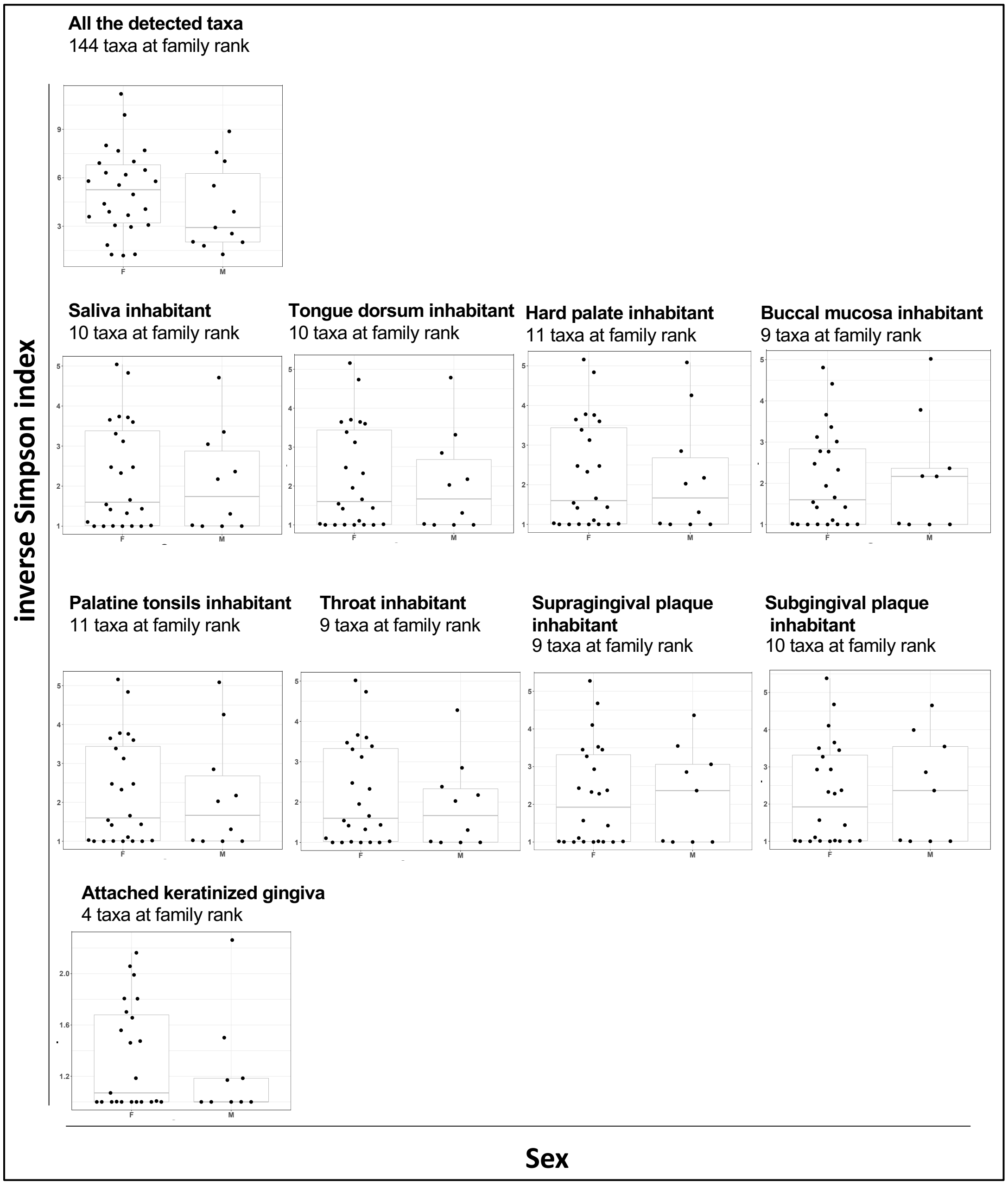

Figure S3. The difference in the inverse Simpson index between sexes evaluated for each set of taxa of inhabitant in the respective oral subsites. The width of the dots' distribution was based on corresponding kernel-density estimation with Gaussian-kernel. Each box denotes interquartile range and each horizontal line in the middle of each box denotes median of corresponding density estimates.

Figure S4

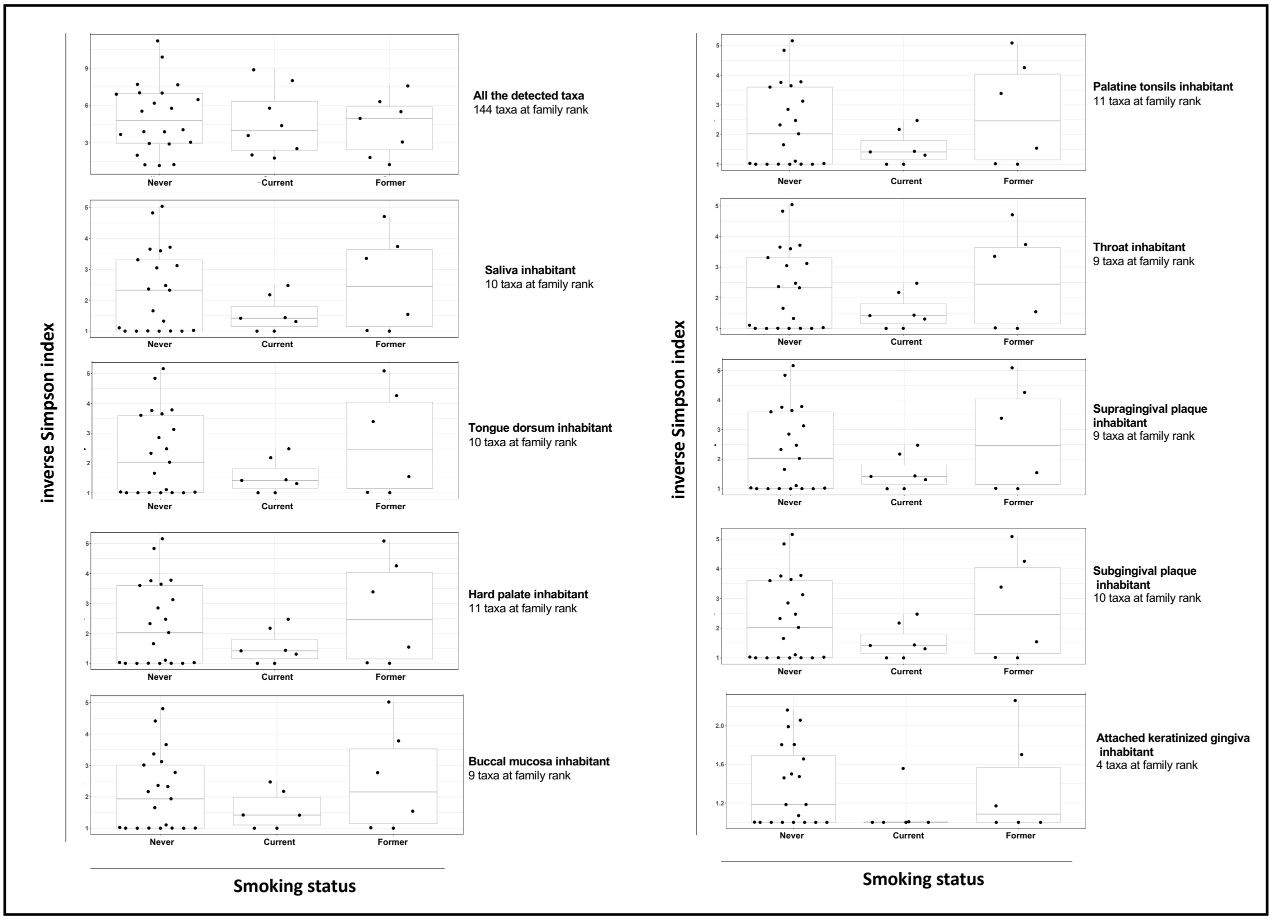

Figure S4. The difference in the inverse Simpson index between smoking status evaluated for each set of taxa of inhabitant in the respective oral subsites. The width of the dots' distribution was based on corresponding kernel-density estimation with Gaussian-kernel. Each box denotes interquartile range and each horizontal line in the middle of each box denotes median of corresponding density estimate.

Figure S5

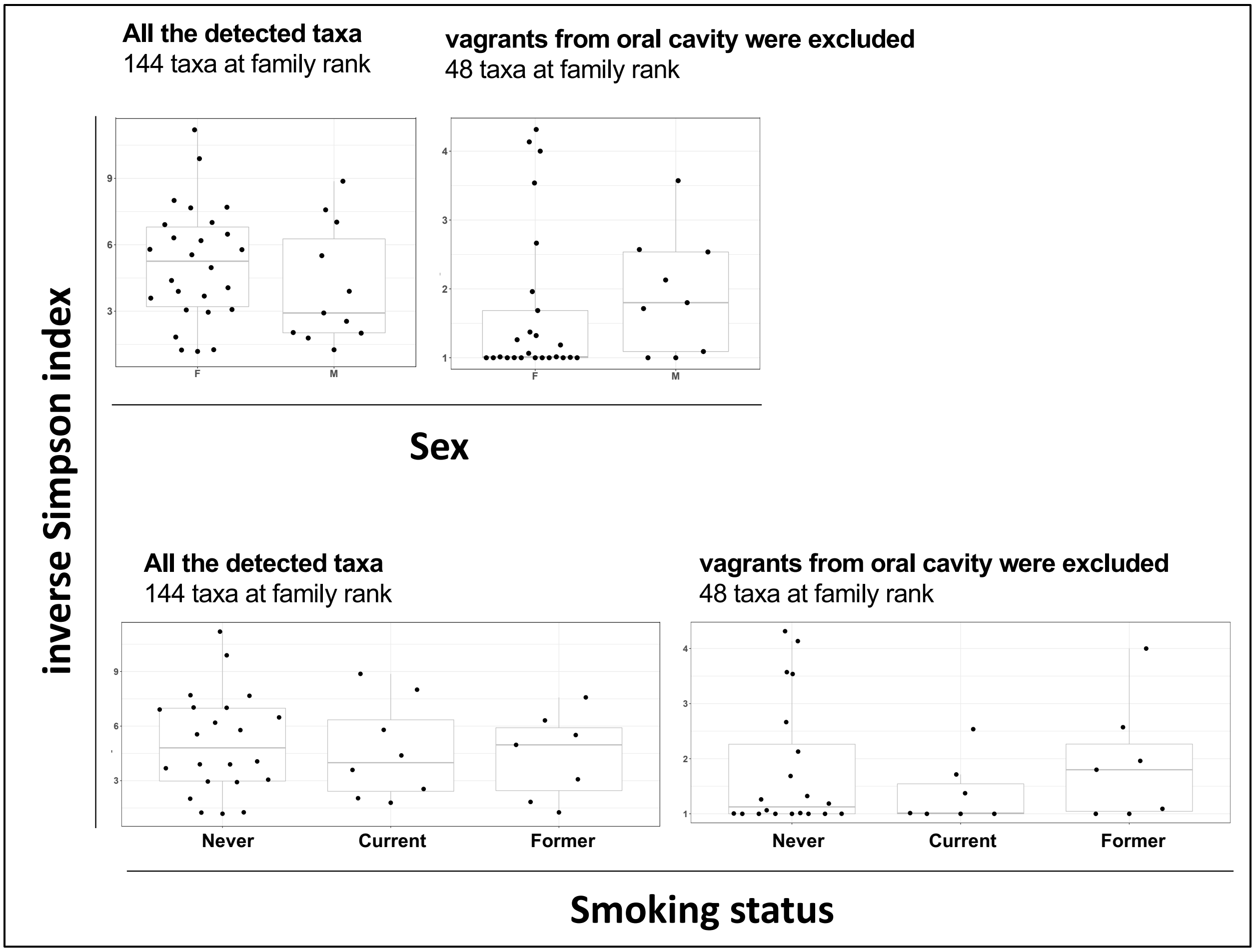

Figure S5. The difference in the inverse Simpson index between sexes and smoking status evaluated for a set of taxa from which the "vagrants" was excluded. The width of the dots' distribution was based on corresponding kernel-density estimation with Gaussian-kernel. Each box denotes interquartile range and each horizontal line in the middle of each box denotes median of corresponding density estimate.

Figure S6

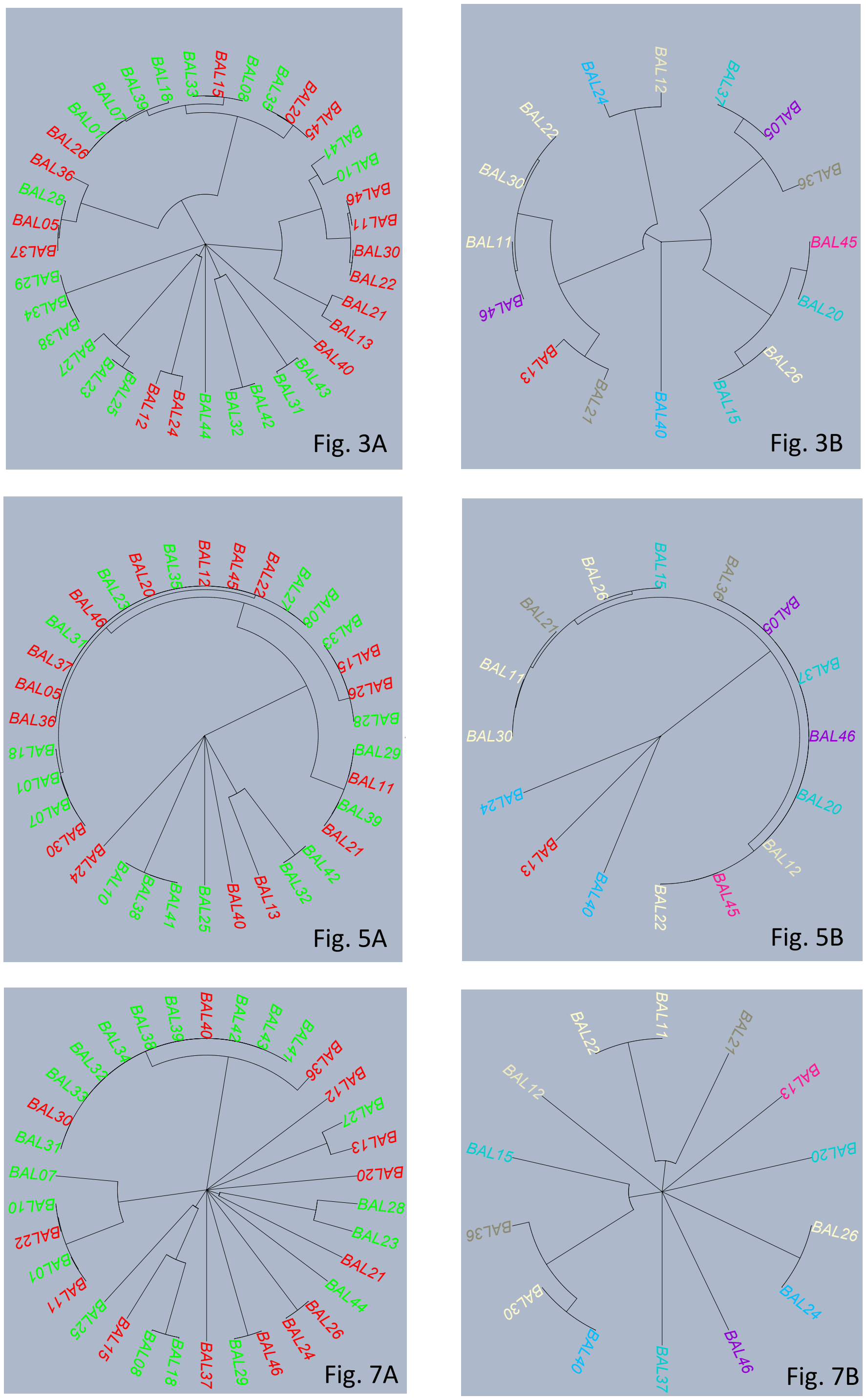

Figure S6. To depict the branching of the clusters more clearly, the dendrograms of the subjects in the Figure 3, 5 and 7 were represented in polar shape.

Figure S7

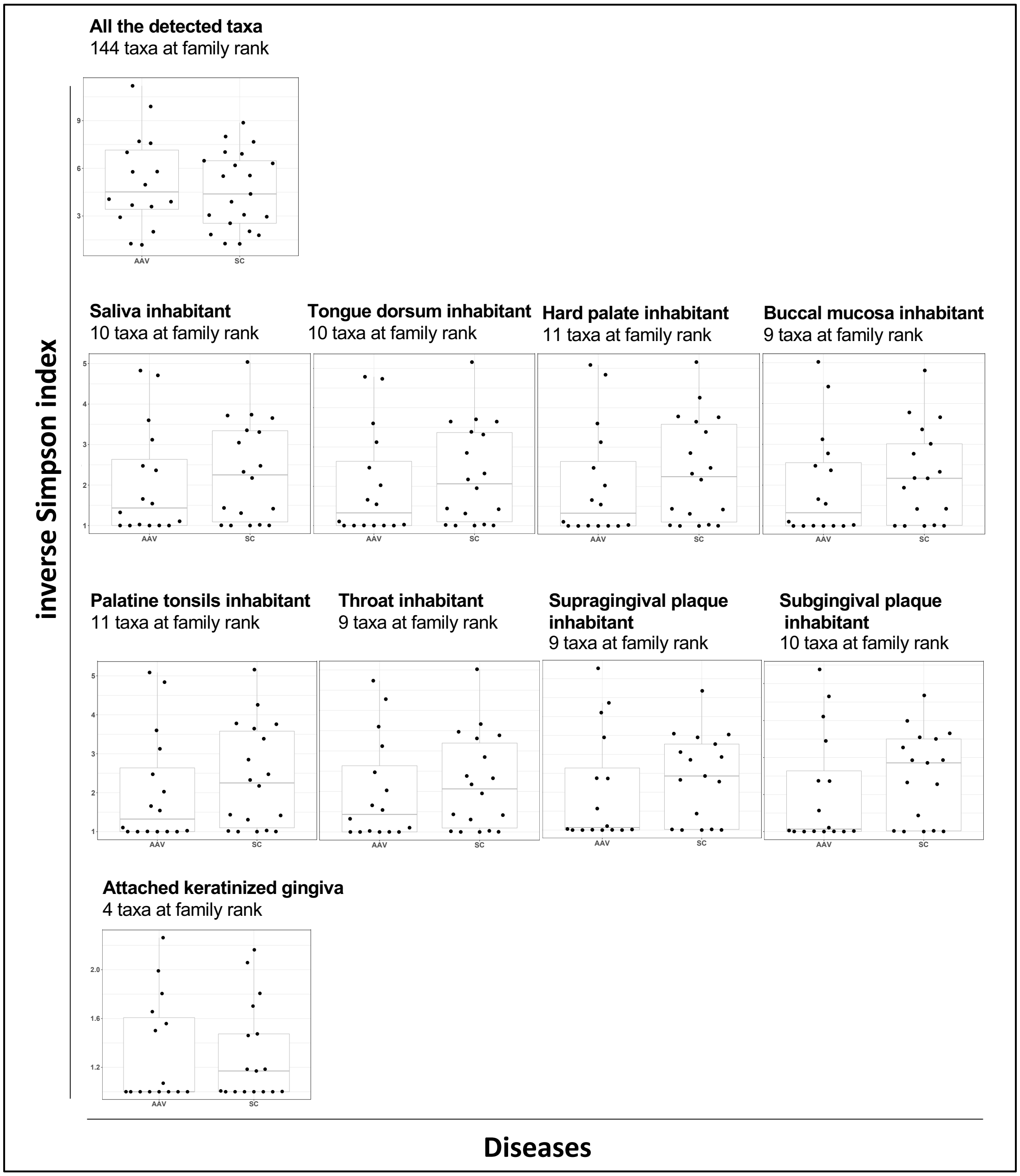

Figure S7. The difference in the inverse Simpson index between diseases evaluated for each set of taxa of inhabitant in the respective oral subsites. The width of the dots' distribution was based on corresponding kernel-density estimation with Gaussian-kernel. Each box denotes interquartile range and each horizontal line in the middle of each box denotes median of corresponding density estimate.

Figure S8

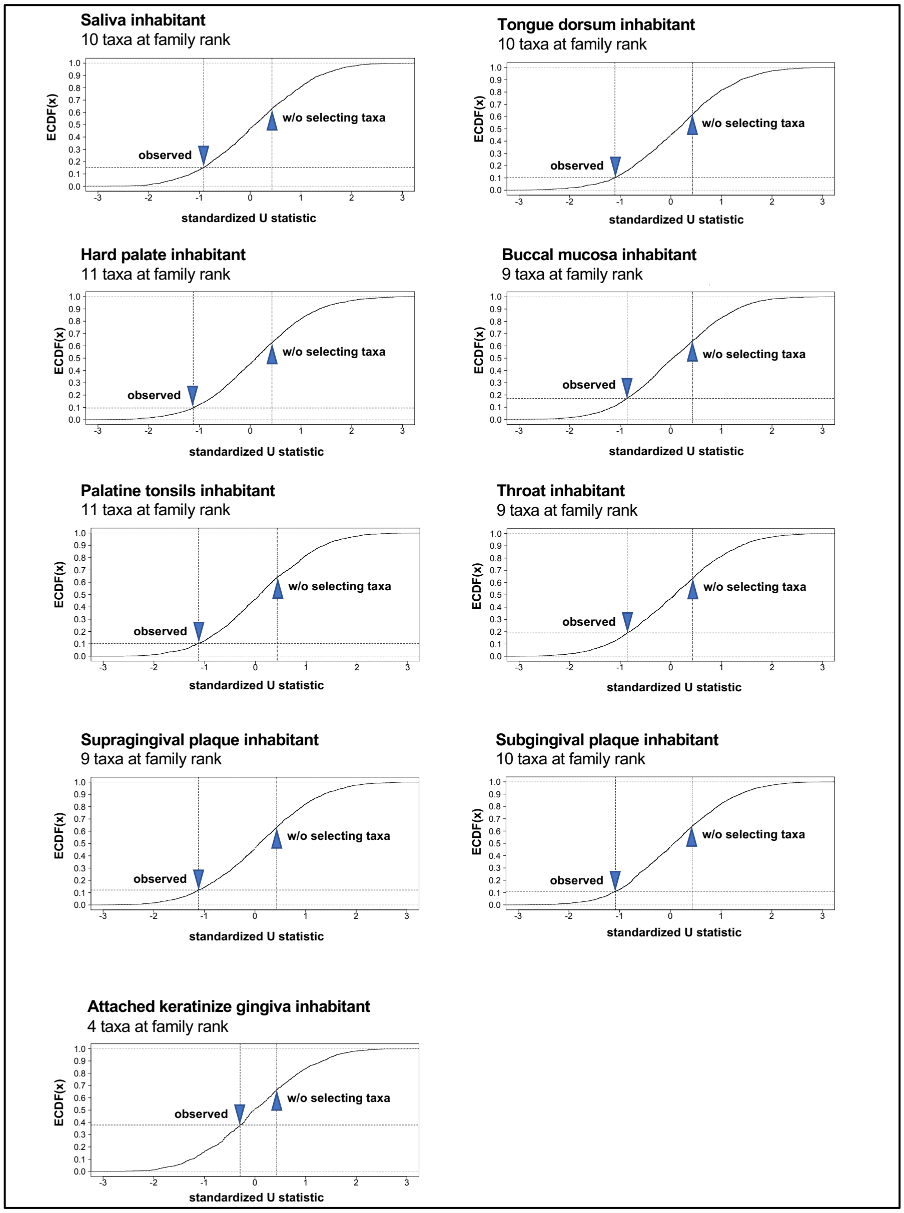

Figure S8. The effect of selecting respective sets of taxa of "inhabitant" contrasted with selecting them in random drawing on the association between the α-diversity and the diseases. The number of the taxa in random drawing were set to the number of taxa in corresponding "inhabitant" for respective bodysites. The standardized U statistics were calculated for the difference in the inverse Simpson index between diseases. The two blue triangles depict the standardized U statistics calculated from the inverse Simpson index for the taxa of "hard palate inhabitant" ("**observed**") and all of the detected taxa ("**w/o selecting taxa**"), respectively.

Figure S9

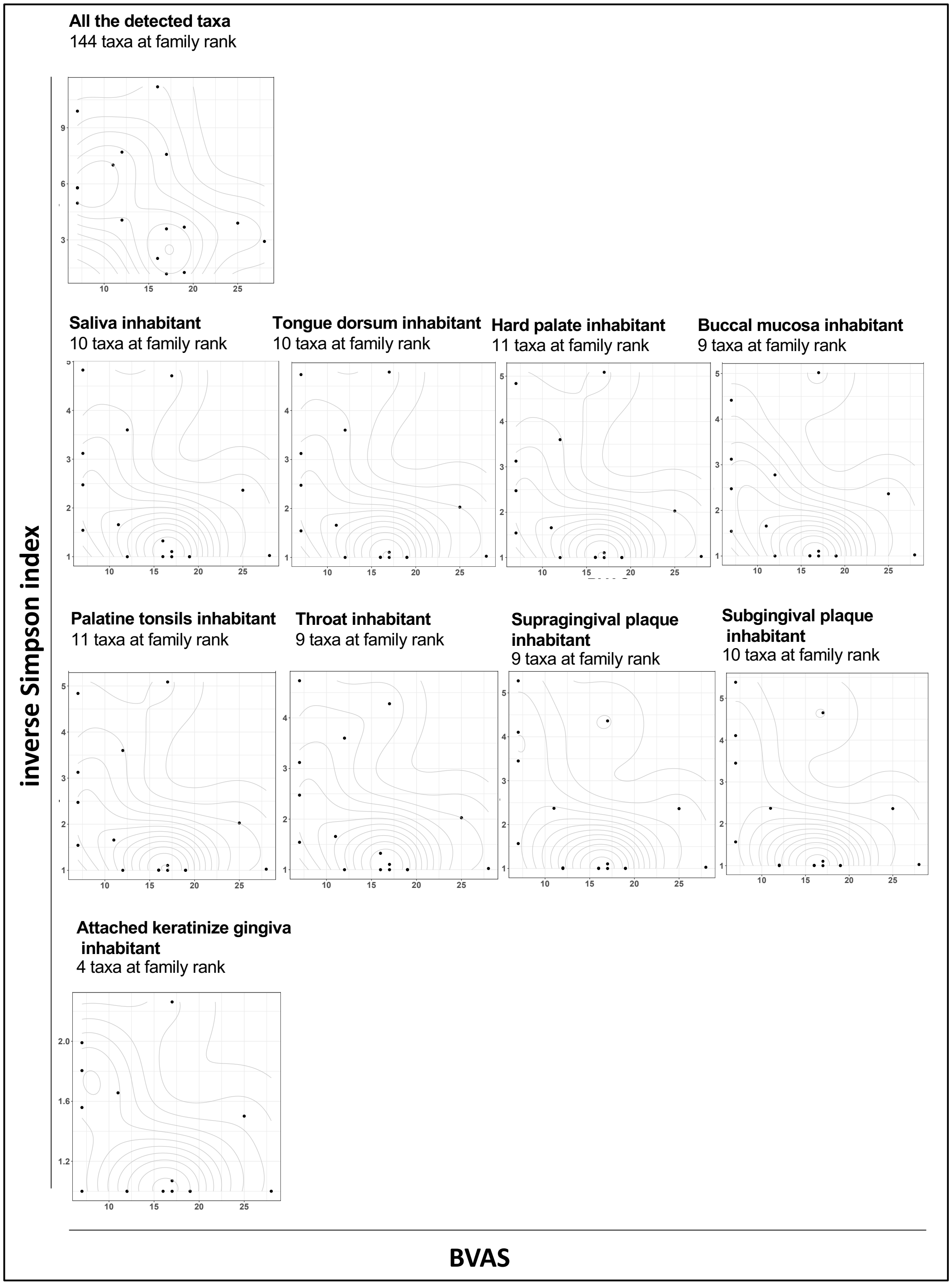

Figure S9. The bivariate relationship between the inverse Simpson index and the BVAS for each set of taxa of inhabitant in the respective oral subsites. The contour lines are two-dimensional probability densities drawn based on corresponding kernel density estimate.

Figure S10

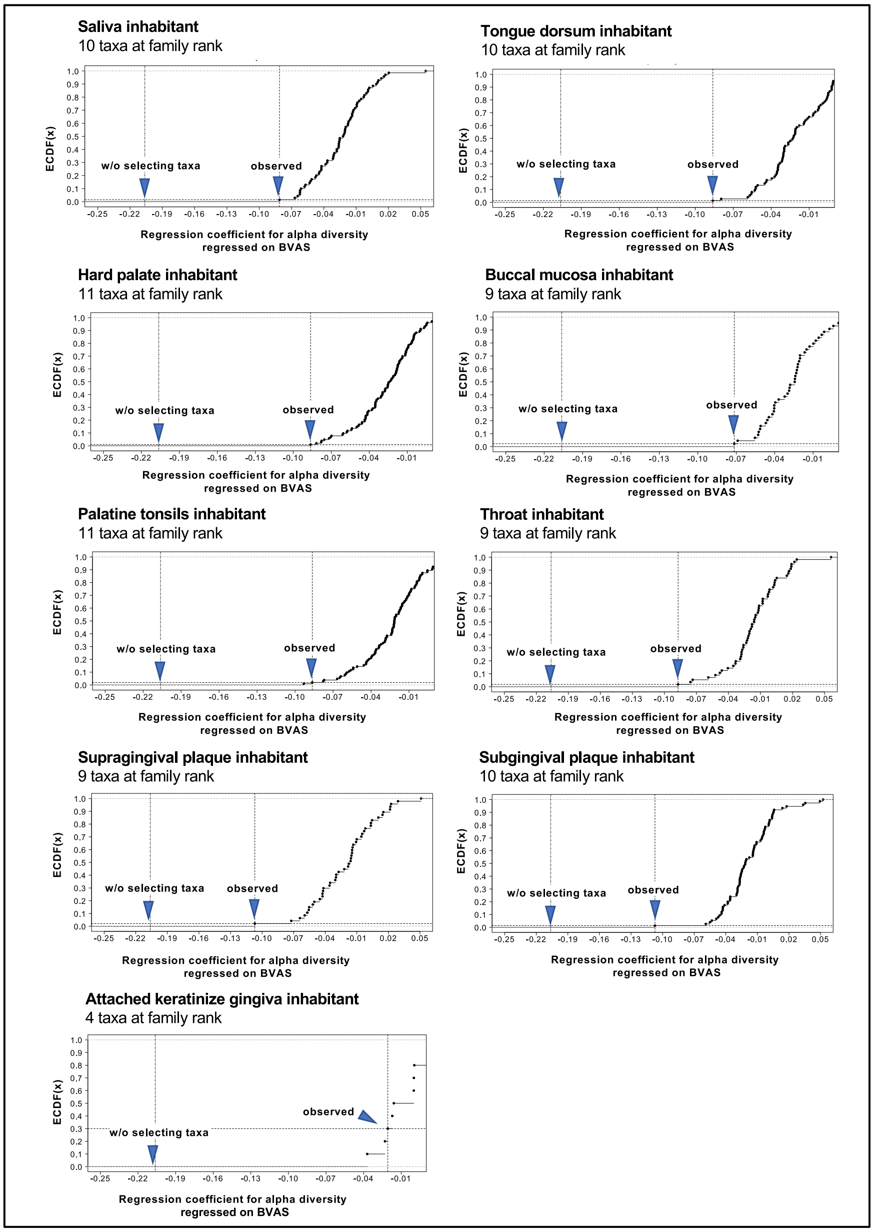

Figure S10. The effect of selecting respective sets of taxa of "inhabitant" contrasted with selecting them in random drawing on the association between the α-diversity and the BVAS. The number of the taxa in random drawing were set to the number of taxa in corresponding "inhabitant". The standardized U statistics were calculated for the difference in the inverse Simpson index between diseases. The two blue triangles depict the standardized U statistics calculated from the inverse Simpson index for the taxa of "hard palate inhabitant" ("**observed**") and all of the detected taxa ("**w/o selecting taxa**"), respectively.
